# Why Does Cognitive Training Yield Inconsistent Benefits? A Meta-Analysis of Individual Differences in Baseline Cognitive Abilities and Training Outcomes

**DOI:** 10.3389/fpsyg.2021.662139

**Published:** 2021-05-26

**Authors:** Hilary J. Traut, Ryan M. Guild, Yuko Munakata

**Affiliations:** ^1^Cognitive Development Center, Department of Psychology & Neuroscience, University of Colorado Boulder, Boulder, CO, United States; ^2^Cognition in Context Lab, Department of Psychology and Center for Mind & Brain, University of California, Davis, Davis, CA, United States

**Keywords:** executive function, episodic memory, compensation effect, magnification effect, cognitive control

## Abstract

Despite growing interest in improving cognitive abilities across the lifespan through training, the benefits of cognitive training are inconsistent. One powerful contributor may be that individuals arrive at interventions with different baseline levels of the cognitive skill being trained. Some evidence suggests poor performers benefit the most from cognitive training, showing compensation for their weak abilities, while other evidence suggests that high performers benefit most, experiencing a magnification of their abilities. Whether training leads to compensation or magnification effects may depend upon the specific cognitive domain being trained (such as executive function or episodic memory) and the training approach implemented (strategy or process). To clarify the association between individual differences in baseline cognitive ability and training gains as well as potential moderators, we conducted a systematic meta-analysis of the correlation between these two variables. We found evidence of a significant meta-correlation demonstrating a compensatory effect, a negative association between initial ability on a trained cognitive process and training gains. Too few papers met our search criteria across the levels of proposed moderators of cognitive domain and training approach to conduct a reliable investigation of their influence over the meta-analytic effect size. We discuss the implications of a compensatory meta-correlation, potential reasons for the paucity of qualifying papers, and important future directions for better understanding how cognitive trainings work and for whom.

## 1. Introduction

There has been steady and growing interest in developing effective methods to manipulate an individual's cognitive abilities. Work on this topic has exploded in recent decades, motivated by rehabilitative and therapeutic goals for impaired or aging populations (Lustig et al., 2009; Dahlin, 2011; van der Donk et al., 2017), by interest in cognitive enhancement for children or the general public (Diamond and Lee, 2011; Diamond, 2012; Simons et al., 2016), as well as by a desire to find means of testing cognitive theories (Cepeda et al., 2001; Chevalier et al., 2014). However, results of cognitive training research have not always been consistent.

While some research finds clear benefits to a trained ability, and even benefits to untrained abilities (both near and far related), other studies yield little to no evidence of benefit from cognitive training. This variation among findings is well-documented and presents serious problems for determining the true effectiveness or utility of cognitive training in both clinical and research contexts (Shipstead et al., 2012; Melby-Lervåg and Hulme, 2013; von Bastian and Oberauer, 2014; Au et al., 2015; Melby-Lervåg et al., 2016; Simons et al., 2016; Rossignoli-Palomeque, 2018). While the design and methodological rigor with which cognitive training research is conducted is an established source of this variation (Noack et al., 2014; Tidwell et al., 2014; Simons et al., 2016; Smoleń et al., 2018) variability remains even when accounting for these concerns. Sources of this remaining variability are of great interest as they could both provide insight into the reasons for the effectiveness or ineffectiveness of cognitive training—illuminating the mechanisms of how trainings work and for whom—and allow for administration of more tailored cognitive trainings in clinical or therapeutic contexts.

Recent evidence suggests that individual differences account for a sizeable amount of the lingering variability in findings over and above methodological differences (Jaeggi et al., 2014; von Bastian and Oberauer, 2014; Borella et al., 2017; Guye et al., 2017). Participants' ages, socio-economic statuses (SES), levels of motivation, and more have all been implicated in influencing the effects of various cognitive trainings (Kliegl et al., 1990; Katz et al., 2014; Segretin et al., 2014, respectively). In particular individual differences in baseline cognitive ability (i.e., an individual's pre-training level of cognitive skill) have been implicated as a prominent source of variation (e.g., Rueda et al., 2005; Jaeggi et al., 2008; Karbach and Unger, 2014; Foster et al., 2017) —suggesting that one way to understand who benefits from what cognitive training is to understand individual abilities upon entering training.

It makes sense that each individual comes into cognitive training with unique abilities, and that baseline could influence effects of training. Multiple theories hypothesize that learning opportunities are most effective when they are tailored to an individual's ability: in child-caregiver interactions (Zone of Proximal Development - Vygotsky, 1978), educational settings (Desirable Difficulty - Bjork and Bjork, 2011), and cognitive interventions (Aptitude by Treatment Interactions - Snow, 1989). Moreover, the influence of other individual difference effects on cognitive training, such as SES and age, appear to be mediated by participants' cognitive baselines (Bürki et al., 2014; Segretin et al., 2014, respectively), suggesting that their influence is actually due in part to baseline cognitive ability. For example, results on individual differences effects of SES suggest that SES does not directly cause differences in cognitive training outcomes, but instead results in a situation that produces different cognitive baseline proficiencies across participants. Given this, one potentially impactful approach to developing a mechanistic understanding of how individual differences give rise to variability in cognitive training outcomes is to focus specifically on individual differences in baseline cognitive ability.

**Figure 1:**
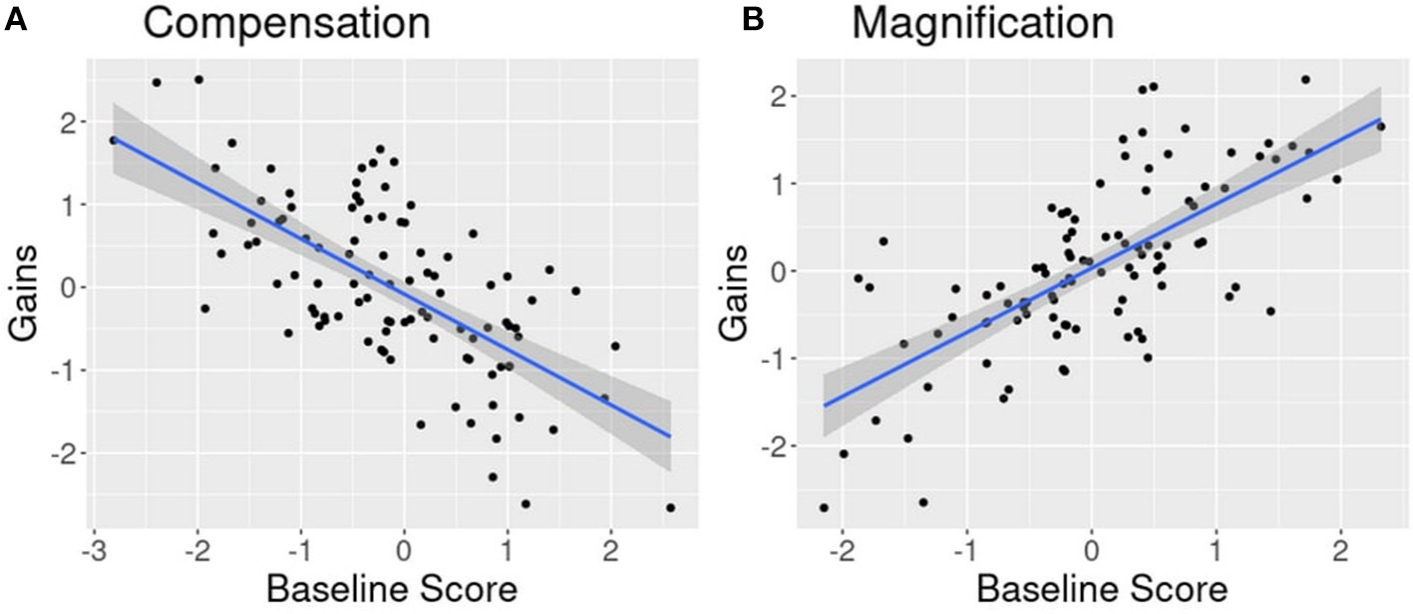
Example of opposing patterns of the relationship between baseline cognitive ability and gains after cognitive training. **(A)** Compensation effect demonstrating lower performing individuals accruing the most gains after training. **(B)** Magnification effect demonstrating higher performing individuals accruing the most gains after training.

However, we do not have a clear picture of the size, or even the direction, of baseline individual differences effects on cognitive training (see Lövdén et al., 2012; Karbach and Unger, 2014; Au et al., 2015, for review). This makes it difficult to get a handle on the importance and informativeness of this phenomenon. Some training studies yield compensation effects: a negative relationship between a participant's initial cognitive ability and the results of training such that worse performing participants demonstrate the greatest benefits from training. In these cases, training appears to allow poor performers to compensate for their weaknesses, such that overall individual differences among participants at pre-training are reduced post-training (Figure 1A). Other training studies yield magnification effects: a positive relationship between participants initial cognitive ability and the results from training. In these scenarios, individuals with the highest levels of performance prior to training experience the most substantial benefits—with individual differences among the training group becoming magnified post-training (Figure 1B).

What might lead to these opposing patterns? Compensation effects may result from a need to master certain skills in order to achieve high levels of performance on a task. These skills may be acquired during training by low performers, bolstering their scores, whereas high performers have already mastered them and receive little benefit (e.g., Lustig et al., 2009). In contrast, magnification effects may result from a certain level of skill being necessary to benefit from a cognitive training intervention. Those who have not reached that threshold do not benefit, while their peers who have reached threshold improve. However, neither of these accounts, laid out in their simplest forms, explain why the literature demonstrates both compensation and magnification effect patterns as opposed to just one or the other.

One clue is that these opposing patterns appear to come from qualitatively different types of cognitive training (see Lövdén et al., 2012; Karbach and Unger, 2014, for review). Most magnification effects come from cognitive training of episodic memory, the ability to retrieve specific information in memory such as the definition of a word or location of a favorite cafe (Kliegl et al., 1990; Verhaeghen et al., 1992; Cox, 1994; Brehmer et al., 2007) (cf. Gaultney et al., 1996; Lövdén et al., 2012). In contrast, most compensation effects come from cognitive training of executive function, abilities related to the control and regulation of behavior (Diamond and Lee, 2011; Zinke et al., 2012, 2014; Karbach et al., 2017) (cf. Loosli et al., 2012; Foster et al., 2017; Wiemers et al., 2019). This raises the possibility that episodic memory and executive function require differing initial levels of proficiency to change with training or that change in these domains develops along different growth functions. For instance, change in episodic memory may necessitate individuals to have already achieved a certain level of proficiency to improve from cognitive interventions. Executive function, on the other hand, may develop in such a way that prior to achieving proficiency there is extensive room for improvement via training—but beyond that point, changes in executive function performance may be more nuanced and difficult to achieve via current training approaches or just difficult to detect in behavioral tasks.

Executive function and episodic memory trainings also differ in the type of training approach utilized, raising the question of whether training approach, and not training domain, determines whether the worst performers or the best performers benefit the most (Lövdén et al., 2012; Karbach and Unger, 2014). Cognitive training of episodic memory primarily utilizes a strategy-based approach, during which participants are given explicit strategy instruction (such as Method of Loci) intended to improve their ability (e.g., Kliegl et al., 1990). Cognitive training of executive function tends to utilize a primarily process-based approach, during which participants practice on one or several related executive function tasks—presumably bolstering their ability implicitly through practice (e.g., Jaeggi et al., 2008). Thus, episodic memory trainings may give rise to magnification effects because explicit strategy instruction could require mastery of an initial skill set. Executive function trainings may give rise to compensation effects because high performers have already acquired the skill set provided through process-based training, leaving them little room to improve compared to less skilled participants still mastering that skill set.

It does not appear that any studies have directly tested the effects of training domain (episodic memory vs. executive function), or tested for an interaction of training domain with training approach (process- vs. strategy-based). To our knowledge, only one study has explicitly investigated questions of training approach (strategy- or process-focused) in baseline individual differences effects on cognitive training, conducting a detailed investigation of change across an episodic memory training by sequentially administering strategy followed by process training manipulations. Analyses showed an initial reduction in individual differences post-strategy instruction (a compensation effect), but magnification of those differences after further process based practice (Lövdén et al., 2012). While these results highlight potential differences in training approach within the episodic memory domain, they are inconsistent with the aforementioned pattern of findings in which strategy training leads to magnification and process training leads to compensation. The design of the study also makes it difficult to rule out the possibility that initial administration of strategy training altered the latter administration of process training via carryover effects. These results highlight that while many studies have tested the association of baseline individual differences with training outcomes, few papers have investigated moderators of this association, leaving many questions unanswered.

Synthesis of investigations into the influence of baseline individual differences is complicated both by conflicting findings and by notable definitional and analysis differences between studies. Studies frequently differ in how they define and operationalize baseline and outcome measures. Precisely what qualifies as “baseline cognitive performance” or “training outcomes” are essential points that change the question ultimately being asked by an analysis. Baseline cognitive ability, for example, is used interchangeably to refer to cognitive skills with varying distance and relation to the exact domain being trained broad and generalized. It could refer to broad cognitive domains operationalized via measures of general cognitive ability or intelligence (e.g., Rueda et al., 2005; Jaeggi et al., 2008; Fu et al., 2017; Hering et al., 2017), but it could also refer to a narrow cognitive domain specific to the to-be-trained cognitive domain, such as a measure of baseline performance on a working memory task administered prior to a working memory training (e.g., Zinke et al., 2012, 2014). Both approaches provide essential information about evaluating who may benefit the most and why, but address conceptually different questions. The former probes how an overall cognitive level may impact training attempts, testing whether a stronger general level of cognitive skill aids training. But, the latter approach asks how ability within the specific cognitive domain or skill targeted by training influences the effects of training.

Near transfer tasks comprise those within the same domain as training but different from the precise task trained (e.g., two different measures of working memory). Far transfer tasks comprise those which measure performance on an ability other than that being trained (e.g., general fluid intelligence performance after working memory training). While some papers address the degree of transfer with regard to training outcomes and individual differences (e.g., Jaeggi et al., 2014), some analyses or discussions do not distinguish between the specific types of training outcomes. This means that some analyses and discussions of baseline individual differences are based on a distant (and potentially tenuous) relationship between cognitive training and far transfer, while others are based on a close relationship between training and final performance on that training. Studies probing this dimension have suggested compensatory effects on trained tasks or near transfer tasks and magnification effects on far transfer tasks (e.g., Borella et al., 2017), (cf. Karbach et al., 2017), emphasizing the importance of being clear about this distinction.

The actual score of training outcomes also takes on many forms—being analyzed as raw training performance during the cognitive training task, scaled training performance (e.g., rank-ordered or z-scored), assignment to low/high performance groups (e.g., based on a median split of performance or another procedure), or calculated gain scores (e.g., change in performance from baseline to post-test). Each of these approaches has unique drawbacks. The use of only raw or scaled training performance to probe the influence of baseline individual differences may merely capture stable individual differences in general ability among participants, as opposed to differences in training outcome specifically. The use of low/high performance groups reduces the power of analyses (Iacobucci et al., 2015). The use of gain scores can lead to uncovering a compensatory association caused by regression to the mean as well as other possible statistical pitfalls (Smoleń et al., 2018).

Analyzing all of these measurement approaches, as opposed to looking at a single means of capturing outcomes, means contending with all of their associated issues. For example, two papers showing conflicting compensation (Karbach et al., 2017) and magnification (Foster et al., 2017) findings on process-based executive function training were derived from studies using very different methods for interrogating individual differences questions. These studies differed both in the characteristics of baseline data analyzed and analysis approach. One paper utilized a continuous measure of each participant's individual performance on baseline and a latent modeling approach (Karbach et al., 2017)—whereas the other changed baseline into a categorical measure, grouping participants into high and low baseline performance groups for analysis combined with ANCOVA procedures (Foster et al., 2017). These differences in capturing baseline performance and analyzing it makes it difficult to compare the two before even considering methodological differences.

Such differences across studies make it difficult to make sense of who benefits most from which kinds of training. Evaluation of any version of questions about individual differences in cognitive baseline and training outcomes is clouded by the likely differing relationships between various combinations of “cognitive baseline” and “training outcomes.” To best leverage individual differences approaches for making theoretical and mechanistic claims about cognitive trainings, we must be clear about what exactly is being analyzed when we look at individual differences.

The current review seeks to provide a systematic synthesis of the literature on individual differences in cognitive baseline and cognitive training outcomes by conducting a planned meta-analysis. A meta-analytic approach to this problem provides several advantages. First, it brings a systematic method to evaluating currently available findings by requiring clear definitions and eligibility criteria to be used in evaluating findings for inclusion in analysis. Second, it leverages the extensive existing work on cognitive training to make inferences. Third, it allows for a powerful investigation of the known heterogeneity of baseline individual differences influences on cognitive training outcomes—turning the methodological diversity of experimental work into an informative tool as opposed to an obstacle to be overcome. Differences in study approaches allow for an investigation into the reliability of this phenomenon as well as possible moderators: cognitive domain trained, training approach, and their interaction. It allows us to test whether accounting for these differences brings clarity to the nature of baseline individual differences association with training outcomes. Lastly, even in the event of null results, a meta-analysis clarifies the existing literature and can produce a more robust synthesis of that literature than non-systematic reviews. This systematic synthesis can then support a more effective design of causal studies to investigate questions surrounding moderators of the association between cognitive baseline and training outcomes.

However, not all of the variability in the literature is helpful, such as the aforementioned variability in definitions and statistical approach. Differences in the variables used in analyses mean that not every study is testing the same type of baseline/outcome association, such that including all of these approaches above in a meta-analysis could obscure relationships. The present review thus focuses the problem space on studies that are comparable in their definitions of cognitive baseline and training outcome, as well as studies that operationalize training outcomes similarly. This was done to limit the introduction of variability unrelated to the theoretical question into the meta-analysis.

First, this review focuses on baseline ability defined as the pre-training performance in the target cognitive domain manipulated by training (e.g., baseline working memory abilities of participants in a working memory training). It therefore excludes studies that define this measure by some general cognitive ability such as fluid intelligence or studies that evaluate baseline individual differences in a cognitive domain different from that being trained. Second, this review constrains outcomes to measures derived from training or near transfer (provided the near transfer task is of the same cognitive domain as training), excluding far transfer outcomes. Far transfer measures could take on an array of forms and vary in how different they are from the trained ability. Currently there is no clear picture of what the relationship between gains in training, near transfer, and far transfer is (Thompson et al., 2013; Au et al., 2015; Sala and Gobet, 2017)—making it unwise to combine all three in the present meta-analysis. Lastly, this review focuses on studies using a consistent form of operationalizing training outcomes as gain scores, as opposed to raw or scaled performance, because this was the most readily available scoring procedure across the literature.

As such, the purpose of this meta-analysis is to (a) examine and synthesize evidence for the association of individual differences with baseline cognitive ability on cognitive training outcomes through correlation coefficients of this relationship and (b) investigate how this correlation is moderated. Specifically, these analyses are designed to answer the following research questions:

1. What is the estimate of the average correlation coefficient of the association between baseline abilities and cognitive training outcomes?
2. Is this correlation moderated by the targeted cognitive domain (i.e., episodic memory or executive function)?
3. Is this correlation moderated by the cognitive training approach (i.e., strategy or practice)?
4. Is this correlation moderated by an interaction between domain and training approach?

## 2. Methods

This review was conducted in accordance with recommendations outlined by the Preferred Reporting Items for Systematic Reviews and Meta-Analyses (PRISMA) group guidelines (Liberati et al., 2009).

### 2.1. Information Sources & Search Protocol

Based on several practical considerations, all literature searches were conducted through Google Scholar. First, a database was sought that allowed for full text searches (i.e., title, abstract, and body of article) due to (a) the diversity of terms utilized in titles, abstracts, and keywords to describe research on cognitive training, and (b) the frequency with which individual differences analyses are discussed only in the body of an article, given that many of these analyses are exploratory or secondary to the primary question of the article. At the time this study was conducted, this restriction limited potential databases for systematic search to Google Scholar or PsychINFO. Google Scholar was selected because it is both an openly accessible and un-curated database. Unlike other “curated” academic databases such as PsychINFO, PubMed, Web of Science, and others—Google Scholar procures search results through an entirely automated algorithmic search of the internet-at-large, as opposed to searching a limited, pre-selected body of entries. While the downside of this approach is that the nature of this algorithm is unknown, this still arguably makes Google Scholar a more unbiased method of searching the literature. Google Scholar is neither beholden to specific database and publisher relationships nor are its searches influenced by curated database add-ons such as enhanced keywords. It also provides the substantial advantage of crawling a diversity of sources that include dissertations, conference proceedings, posters, unpublished work, and similar “gray” literature because of its broad un-curated search approach. Therefore, the use of Google Scholar helps to circumvent potential issues resulting from both a lack of common terminology in titles, abstracts, and keywords, as well as issues resulting from bias in search results attributed to the curated and financial nature of curated database services.

As such, all searches were conducted in Google Scholar utilizing the following protocol:

“cognitive training” AND “individual differences”

While the use of such a broad protocol in a full-text search was expected to produce a large initial pool of studies, it was selected to ensure the capture of the majority of studies likely to contain relevant data in order to provide as unbiased a review of the available data as possible. The search protocol was constrained to the years 1980 to 2018 (inclusive) to best capture the modern literature on cognitive training. Searches were conducted within specific year intervals (e.g., 1999 to 1999) to address Google Scholar's 1,000 results limit. This allowed for access to the complete set of articles determined by Google Scholar's search. For individual years with results exceeding 1,000 articles, the search protocol was separated into two parts by searching for articles based on the required inclusion or exclusion of a common word, “baseline”:

“cognitive training” AND “individual differences”

AND “baseline”

“cognitive training” AND “individual differences”

NOT “baseline”

Returned search results were extracted from Google Scholar results pages using an automated script implemented in Python (see Supplementary Materials). This script extracted title, author, journal, digital access (e.g., website), and related publication information. Complete abstracts for each search result were extracted manually by trained research assistants and saved in a complete document of all search results and retrieved information as Google Scholar search results do not return complete abstracts. All information was recorded in a single tracking document.

### 2.2. Eligibility Criteria

Eligibility was determined by the first author of this paper (H.J. Traut) over the course of unblinded reviews of retrieved search results. Eligibility criteria were designed along the “PICOS” recommendations of the PRISMA guidelines (Liberati et al., 2009) to account for Participants, Intervention, Comparators, Outcome, and Study design. Additional categories of criteria were included as necessitated by the demands of the present systematic review.

#### 2.2.1. Participants

Studies were not excluded based on the nature of participants provided that participants were human (e.g., excluding cognitive training studies conducted with rodents or other common animal subjects). Participants of any age, neurological status, or other characteristics were eligible for inclusion. This decision was made to allow for the maximum number of results to be retrieved and due to the absence of hypotheses explicitly regarding differences in baseline individual difference effects between typical and atypical individuals.

#### 2.2.2. Intervention (i.e., training)

To be eligible, studies were required to include experiment(s) in which at least one group of participants underwent training with a task intended to manipulate the participants' executive function (EF) or episodic memory (EP) abilities according to the following definitions:

**Episodic Memory Training**: Training intended to manipulate an individual's ability to retrieve explicit information such as facts, definitions, or experiences (Tulving, 1972).

**Executive Function Training**: Training intended to manipulate an individual's ability to control and regulate their behavior or the components of that ability, such as inhibition, updating, and shifting (Miyake et al., 2000; Miyake and Friedman, 2012).

Examples of episodic memory training studies would include mnemonic trainings such as Method of Loci (e.g., Cox, 1994). Examples of executive function training studies would include many working memory or inhibition trainings (e.g., von Bastian and Oberauer, 2014). Studies including training on both executive function and episodic memory were excluded as they would not allow for differentiation of the theorized interaction effect between training domain and the influence of baseline individual differences on outcomes.

Trainings were further required to consist of a task or procedure that directly involved a cognitive skill and was either a strategy or process-based training following the definitions provided by Lustig et al. (2009):

**Strategy Training**: The explicit instruction of a strategy with the intent of helping participants improve their performance on the target domain.

**Process Training**: The administration of either a single task or set of tasks directly derived from or related to the target domain for participants to practice with.

Studies including trainings that integrated both strategy and process elements were excluded because the inclusion of both approaches would make it impossible to dissociate their effects in the proposed moderator analysis. Similarly, studies administering process or strategy-based trainings alongside additional manipulations were also excluded (i.e., multi-modal interventions). This included, but was not limited to, trainings with manipulations such as cardio-vascular exercise, mindfulness, commercial/popular video-games (e.g., World of Warcraft), social skills training, and emotional skills training. It also included training interventions that involved administration of neurostimulation, neurofeedback, pharmacological interventions, therapeutic interventions (such as cognitive behavioral therapy), or other concurrent additional manipulations to cognitive training. These exclusions were included to ensure that analyzed effects could reasonably be assumed to result from cognitive training manipulations alone and not the influence of additional manipulations not of interest.

#### 2.2.3. Comparator

Studies were required to include at least one eligible treatment group based on the criteria described above. They were not required to include a comparison group of any kind. If present, the type of comparison group was recorded for all eligible studies (e.g., active or passive controls).

#### 2.2.4. Baseline & Outcome Measures

Studies were required to have measured participants baseline ability in the trained cognitive skill(s) prior to training. For example, if a study tested the effects of a working memory training, a baseline measure of working memory ability prior to training was required for inclusion. This baseline measure could come from the same task that was later used for training or from a different task that assessed the same cognitive skill. Studies were required to have measured participants cognitive training outcomes using gain scores. Gain scores were defined as any calculation of the difference in performance between baseline performance and final performance. To be eligible these outcomes needed to measure the trained cognitive skill(s), and were thus derived from either final training performance or performance on a near transfer task.

#### 2.2.5. Study Design

Studies were required to include a pre- and post- design such that there were measurements of participants performance before and after the cognitive training. Studies needed to be experimental or quasi-experimental in nature. Observational studies, case studies, case-series, qualitative studies, or studies not using a pre-/post- design were excluded.

#### 2.2.6. Other

Studies were not excluded for administration of multiple types of cognitive training across multiple groups, provided that at least one cognitive training group met the above criteria. No exclusion criteria were imposed based on the country of origin, but only papers for which a full-text English version of the article was available were evaluated for eligibility.

### 2.3. Study Selection

After search results were collected using the search protocol described above, results were saved in a systematized electronic format to be reviewed for eligibility. Prior to review, duplicate papers were flagged through the use of an automated script. To confirm automated results, the first author manually verified duplication of flagged entries.

Post-duplication flagging, results were scanned for inclusion based on titles and abstracts. Results were flagged and removed at this stage if they were obviously ineligible (i.e., pertaining to unrelated topic or clearly not conducting a cognitive training intervention) or failed to meet eligibility criteria (e.g., subjects were not human). Full text documents were obtained for remaining articles. Each full-text document was screened for eligibility based on the established eligibility criteria discussed above. Results are reported below.

### 2.4. Data Collection & Coding

All papers that passed the eligibility screening processes were coded for relevant data. These data included number of training groups, sample size (broken down by training group), cognitive training characteristics (including trained domain, training approach, training tasks, etc.), and relevant statistical analyses. From the relevant statistical analyses, baseline measures and outcome measures were recorded. Data were also coded for participant characteristics (e.g., age(s), neurological status, population). Coders were not masked to the article's authors, institution, or place of publication.

### 2.5. Correlation Coefficient Extraction

Correlations between baseline ability and gain scores were selected for meta-analysis as this presented the most readily available effect size measure across retrieved studies. Studies were allowed to contribute multiple correlation coefficients if more than one was provided. For example, if a study reported multiple eligible correlation coefficients assessing the relationship between cognitive baseline and training outcomes—all of these correlations were recorded. This was done to allow the maximum amount of observations to be included in meta-analysis as well as to avoid introducing bias by determining an *ad-hoc* method of selecting a single effect size from each study for inclusion. Study is included as a level in our multi-level model as described below.

### 2.6. Analysis Plan

After extraction, correlation coefficients were scaled using a Fisher's Z transformation (Lipsey and Wilson, 2001). Publication bias was evaluated using Egger's regression test and visual inspection for funnel plot asymmetry. This permitted evaluation of the degree to which the characteristics of the funnel plot deviate from normal based on their precision (Egger et al., 1997). Heterogeneity between studies was evaluated using both Cochrane's Q and *I*^2^ measures. If a significant amount of heterogeneity was observed, initial analyses were followed with those described below.

#### 2.6.1. Model 1: Meta-Analytic Correlation

To estimate the mean association between individual differences in baseline and cognitive training gains, a three-level meta-analytic model was implemented. A multi-level model was selected to allow for multiple correlation coefficients to be clustered within a study to both account for similarities between those correlations inherent to having been collected from the same study and control for the possibility of one study with multiple significant observations from over-contributing to results (Konstantopoulos, 2011; Van den Noortgate et al., 2013; Cheung, 2014). This approach also incorporates weighting each observation in analysis according to its respective sensitivity—i.e., sample size—such that effect sizes derived from larger samples contributed more to analysis (Borenstein et al., 2010). The complete model therefore included random effects of both study (Level 3) and correlation coefficient (Level 2). Level 3 captured variance between studies in which effect sizes were clustered. Level 2 captured variance among the effect sizes within these studies. Level 1 captured the sampling variance of the actual correlation coefficient effect sizes themselves. This model is outlined in Equation (1), where *i* refers to individual correlation coefficient observation, *j* refers to individual studies. κ refers to the average correlation coefficient within study *j*, β refers to the average population level correlation coefficient, θ refers to true correlation coefficient, and $\hat{\theta }$ refers to the estimate of observation *i* within study *j*.

(1) $$\begin{array}{l} \begin{array}{c} Level1:{\hat{\theta }}_{ij}=\theta_{ij}+\epsilon_{ij}\\ Level2:\theta_{ij}=\kappa_{j}+\xi_{\left(2\right)ij}\\ Level3:\kappa_{j}=\beta_{0}+\xi_{\left(3\right)j} \end{array} \end{array}$$

#### 2.6.2. Model 2: Meta-Regression

To evaluate the prediction that training approach and trained domain moderate the association between baseline ability and training gains, a second three-level mixed effects meta-analytic model was planned. Levels 1 and 2 were identical to those of Model 1. At Level 3, fixed effects for training approach (strategy or process) and trained domain (episodic memory or EF) were included as predictors.

(2) $$\begin{array}{l} \begin{array}{l} Level1:{\hat{\theta }}_{ij}=\theta_{ij}+\epsilon_{ij}\\ Level2:\theta_{ij}=\kappa_{j}+\xi_{\left(2\right)ij}\\ Level3:\kappa_{j}=\beta_{0}+\beta_{1}\left(CognitiveDomain\right)+\\ \beta_{2}\left(TrainingApproach\right)+\xi_{\left(3\right)j} \end{array} \end{array}$$

Both training approach and trained domain predictors were dummy coded.

## 3. Results

### 3.1. Search Results

**Figure 2:**
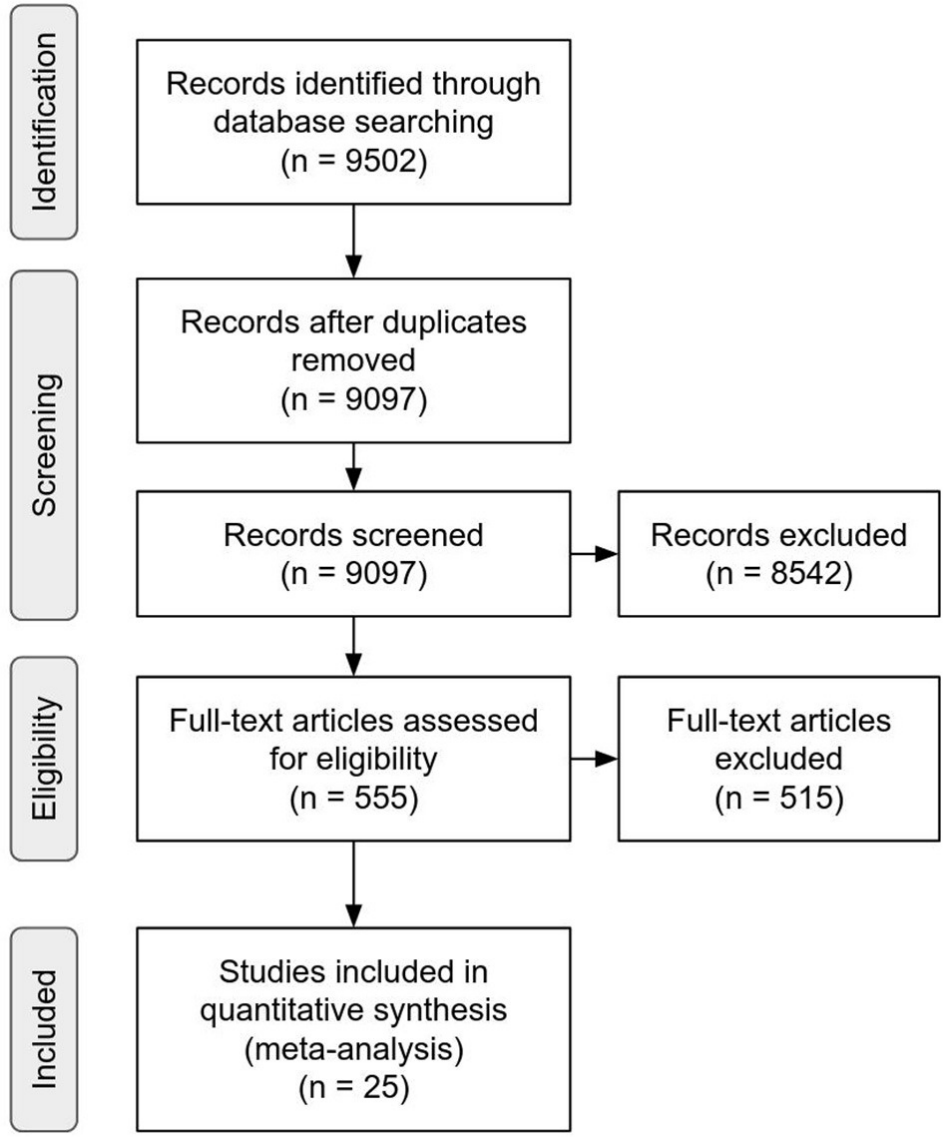
PRISMA 2009 flowchart depicting literature retrieval process from initial database querying to studies included in analysis.

The initial search protocol within Google Scholar (run between November–December 2018) produced 9,502 documents, of which 405 were identified as duplicates. During the screening of the remaining 9,097 unique documents abstracts and titles, 8,542 documents were excluded. The remaining 555 underwent full text screening for eligibility. A total of 42 papers met the eligibility criteria described above. Of these, 15 reported sufficient information for extraction or calculation of a correlation coefficient. Twenty-seven papers met eligibility criteria but did not report sufficient information for calculation of relevant effect sizes. Authors of these papers were contacted and requested to provide either the requisite correlation or the raw data necessary to calculate the requisite correlations. Ten papers were added via this contact procedure for a final sample of 25 papers (Figure 2; Table 1). All reported meta-analytic tests were conducted based on a total of 82 correlation coefficients extracted from this set of papers reporting correlation coefficients (see Table 1). A range of 1 to 8 correlation coefficients were reported in each paper with an average of 2.5. One study (Lövdén et al., 2012) conducted a two-part training consisting of a strategy approach followed by a process approach. Eligible correlation coefficients were presented after each training, so correlation coefficients presented after the initial strategy training are included whereas the post-process training data are excluded. Of retrieved papers, 3 were episodic memory trainings and 22 were executive function trainings (including 18 working memory, 2 inhibition, 1 task switching, and 1 selective attention). There was a 1:1 ratio of cognitive domain and training approach such that all 3 episodic memory trainings were strategy-based and all 22 executive function trainings were process-based approaches.

**Table 1:** List of all observations used in reported analyses.

**Author(Year)**	**Correlation ID**	**Training domain**	**Training approach**	***n***	***r***
Chan et al. (2015)	1	EF	Process	25	–0.72
”	2	EF	Process	25	–0.51
”	3	EF	Process	25	–0.50
”	4	EF	Process	25	–0.81
”	5	EF	Process	25	–0.17
”	6	EF	Process	25	–0.30
”	7	EF	Process	25	–0.53
”	8	EF	Process	25	–0.37
Chooi (2011)	1	EF	process	9	-0.04
”	2	EF	Process	13	–0.48
Fellman et al. (2018)	1	EF	Process	25	–0.62
Foster et al. (2017)	1	EF	Process	30	–0.26
”	2	EF	Process	30	–0.15
”	3	EF	Process	36	–0.34
”	4	EF	Process	36	0.06
Gade et al. (2017)	1	EF	Process	10	–0.86
”	2	EF	Process	16	–0.71
”	3	EF	Process	10	–0.95
”	4	EF	Process	10	–0.74
Gunn et al. (2018)	1	EF	Process	70	–0.01
Hickey (2018)	1	EF	Process	5	0.05
”	2	EF	Process	5	0.70
Jones et al. (2020)	1	EF	Process	41	0.34
Karbach et al. (2017)	1	EF	Process	126	0.81^*^
Karbach et al. (2015)	1	EF	Process	14	–0.39
Lövdén et al. (2012)	1	EP	strategy	50	–0.85
”	2	EP	Strategy	29	–0.99
”	3	EP	Strategy	29	–0.90
”	4	EP	Process	50	0.33
”	5	EP	Process	29	–0.02
”	6	EP	Process	29	–0.09
McKitrick et al. (1999)	1	EP	Strategy	224	–0.33
”	2	EP	Strategy	224	–0.44
O'Brien et al. (2013)	1	EF	Process	11	–0.63
De Simoni and von Bastian (2018)	1	EF	Process	59	–0.4
”	2	EF	Process	59	–0.26
”	3	EF	Process	59	–0.52
”	4	EF	Process	59	–0.58
”	5	EF	Process	66	–0.28
”	6	EF	Process	66	–0.48
”	7	EF	Process	65	–0.15
”	8	EF	Process	66	0.05
Singer et al. (2003)	1	EP	Strategy	96	0.14
Stepankova et al. (2014)	1	EF	Process	20	–0.01
”	2	EF	Process	20	0.02
Strobach and Huestegge (2017)	1	EF	Process	76	0.15
”	2	EF	Process	76	0.16
”	3	EF	Process	76	0.40
”	4	EF	Process	76	0.39
”	5	EF	Process	76	0.24
”	6	EF	Process	76	–0.47
Vermeij et al. (2017)	1	EF	Process	21	0.03
”	2	EF	Process	21	–0.58
”	3	EF	Process	20	–0.63
”	4	EF	Process	20	–0.39
”	5	EF	Process	14	–0.16
”	6	EF	Process	14	–0.30
”	7	EF	Process	10	–0.22
”	8	EF	Process	10	–0.50
Volckaert and Noël (2015)	1	EF	Process	24	–0.55
Volckaert and Pascale Noel (2017)	1	EF	Process	16	–0.72
von Bastian and Oberauer (2013)	1	EF	Process	30	–0.27
”	2	EF	Process	30	–0.54
”	3	EF	Process	31	–0.79
de Vries et al. (2018)	1	EF	Process	81	0.51
Weicker et al. (2018)	1	EF	Process	20	–0.13
”	2	EF	Process	20	–0.25
”	3	EF	Process	20	–0.60
”	4	EF	Process	20	–0.31
”	5	EF	Process	20	–0.63
”	6	EF	Process	20	–0.59
”	7	EF	process	20	–0.53
”	8	EF	Process	20	–0.55
”	9	EF	Process	19	–0.56
Zinke et al. (2012)	1	EF	Process	20	–0.66
”	2	EF	Process	20	–0.59
”	3	EF	Process	20	–0.65
”	4	EF	Process	20	–0.89
”	5	EF	Process	20	–0.80
Zinke et al. (2014)	1	EF	Process	40	–0.46
”	2	EF	Process	40	–0.17
”	3	EF	Process	40	–0.32
”	4	EF	Process	40	0.10
”	5	EF	Process	40	–0.64
”	6	EF	Process	40	–0.30

*EF, executive function; EP, episodic memory*.

* *Indicates correlation was multiplied by -1 for interpretability purposes in analysis*.

### 3.2. Analysis Results

All analyses were carried out using the R Opensource Framework, R version 3.6.0, (R Core Team, 2020) and the metafor package (Viechtbauer, 2010; Assink and Wibbelink, 2016). Correlation coefficients were standardized via Fisher's Z transformation (Lipsey and Wilson, 2001). For extended analysis reports see Supplementary Materials.

#### 3.2.1. Bias

**Figure 3:**
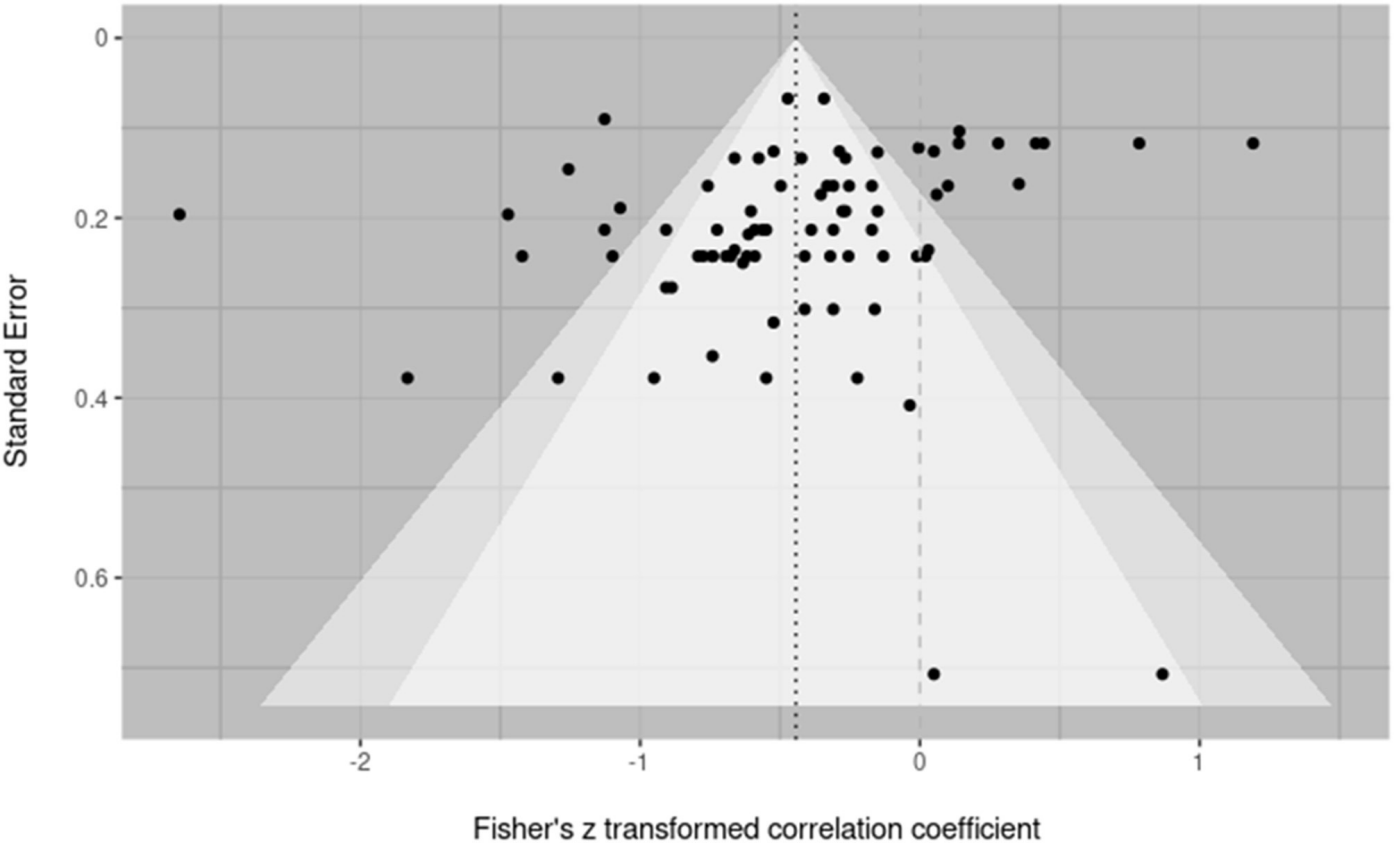
Contoured funnel plot of all effect sizes. Fisher's Z transformed correlation coefficients for each reported effect size are plotted against the standard error. The dark dashed line indicates the summary effect size on which the funnel is centered. The lighter dashed line indicates a null effect. The funnel is centered on the summary effect size. Egger's regression suggests mild asymmetry present in the plot (z = –1.7653; *p* = 0.0775).

**Figure 4:**
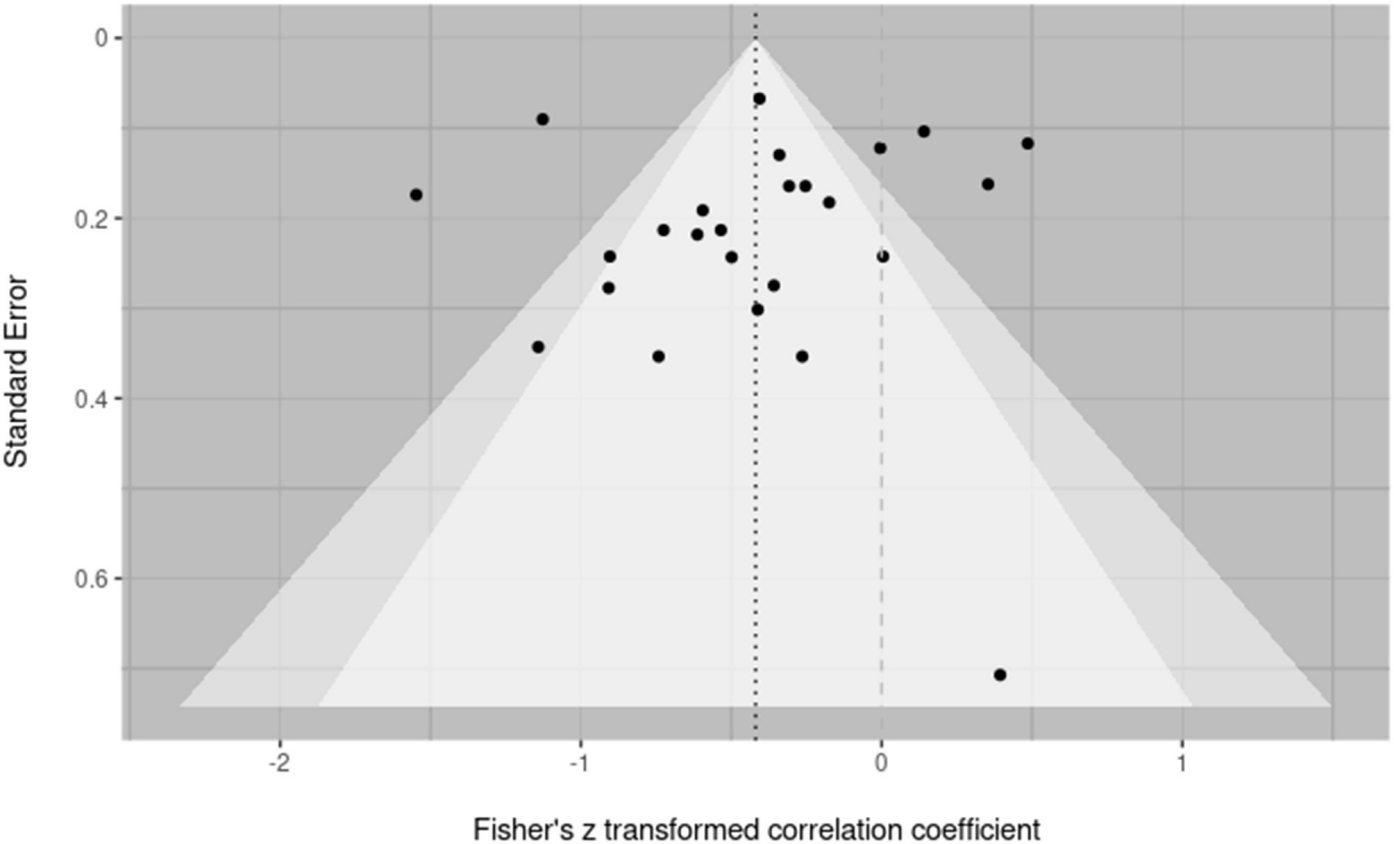
Contoured funnel plot of effect sizes aggregated by paper. Fisher's Z transformed correlation coefficients for each reported effect size are plotted against the standard error. The dark dashed line indicates the summary effect size on which the funnel is centered. The lighter dashed line indicates a null effect. The funnel is centered on the summary effect size. Egger's regression suggests no asymmetry present in the plot (z = -0.4545, *p* = 0.6495).

Publication bias was assessed over both individual effect sizes and averaged effect sizes within a study. This was done to most accurately reflect the inclusion of multiple effect sizes from within a single study and adhere to the purpose of bias evaluation methods to be conducted over individual studies (Fernández-Castilla et al., 2020). In both cases, bias was assessed by visual inspection of a funnel plot and testing for funnel plot asymmetry using Egger's regression. Across all effect sizes, Egger's regression suggested asymmetry was present within the corresponding funnel plot at the recommended threshold of below *p* = 0.10 (z = –1.7653; *p* = 0.0775) (Egger et al., 1997). Visual inspection suggested two small n effect sizes in the lower right (both from the paper Hickey, 2018) might be the source of this asymmetry (see Figure 3). Evaluation of bias across effect sizes aggregated within study would suggest this is the case. The aggregated effect size funnel plot (Figure 4) shows little indication of asymmetry with visual inspection, revealing a more symmetrical funnel. Egger's regression correspondingly returns non-significant findings for asymmetry (z = –0.4545, *p* = 0.6495). Variability in the upper strata along the x axis suggests notable heterogeneity among observations, which is supported by heterogeneity analyses discussed below, and is not in-and-of itself indicative of asymmetry or bias (Sterne et al., 2011).

#### 3.2.2. Model 1: Meta-Analytic Correlation

**Figure 5:**
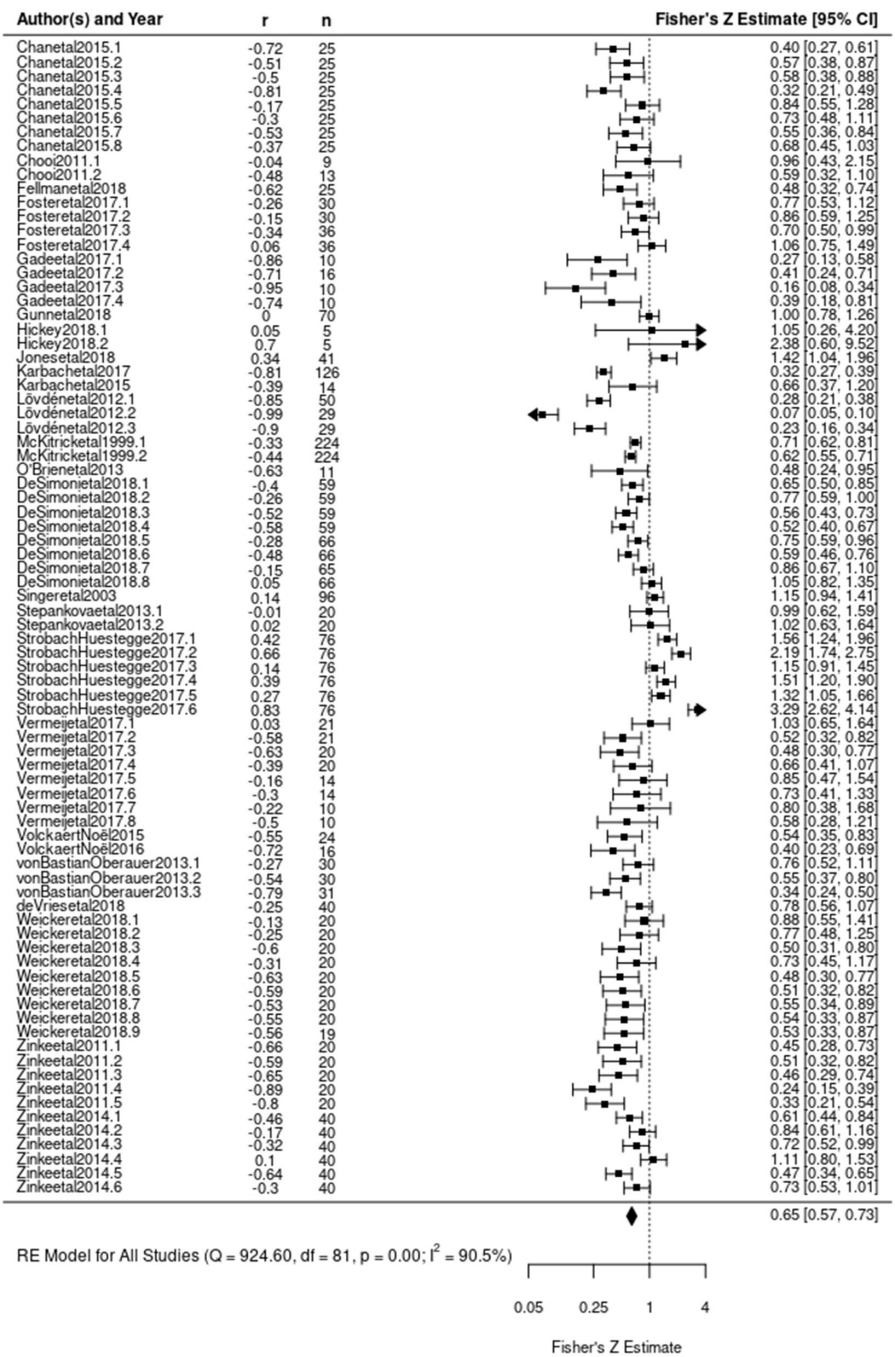
Forest plot of Model 1 results. Each correlation coefficient is plotted according to its Fisher's Z transformation. Size of square indicates weight assigned to the observation during analysis. Length of lines extending from square indicates variance associated with observation based on sample size. Each observation's Fisher's Z transformation and confidence interval are presented on the right. The diamond at the bottom indicates the estimated Fisher's Z transformed correlation coefficient derived from Model 1.

**Figure 6:**
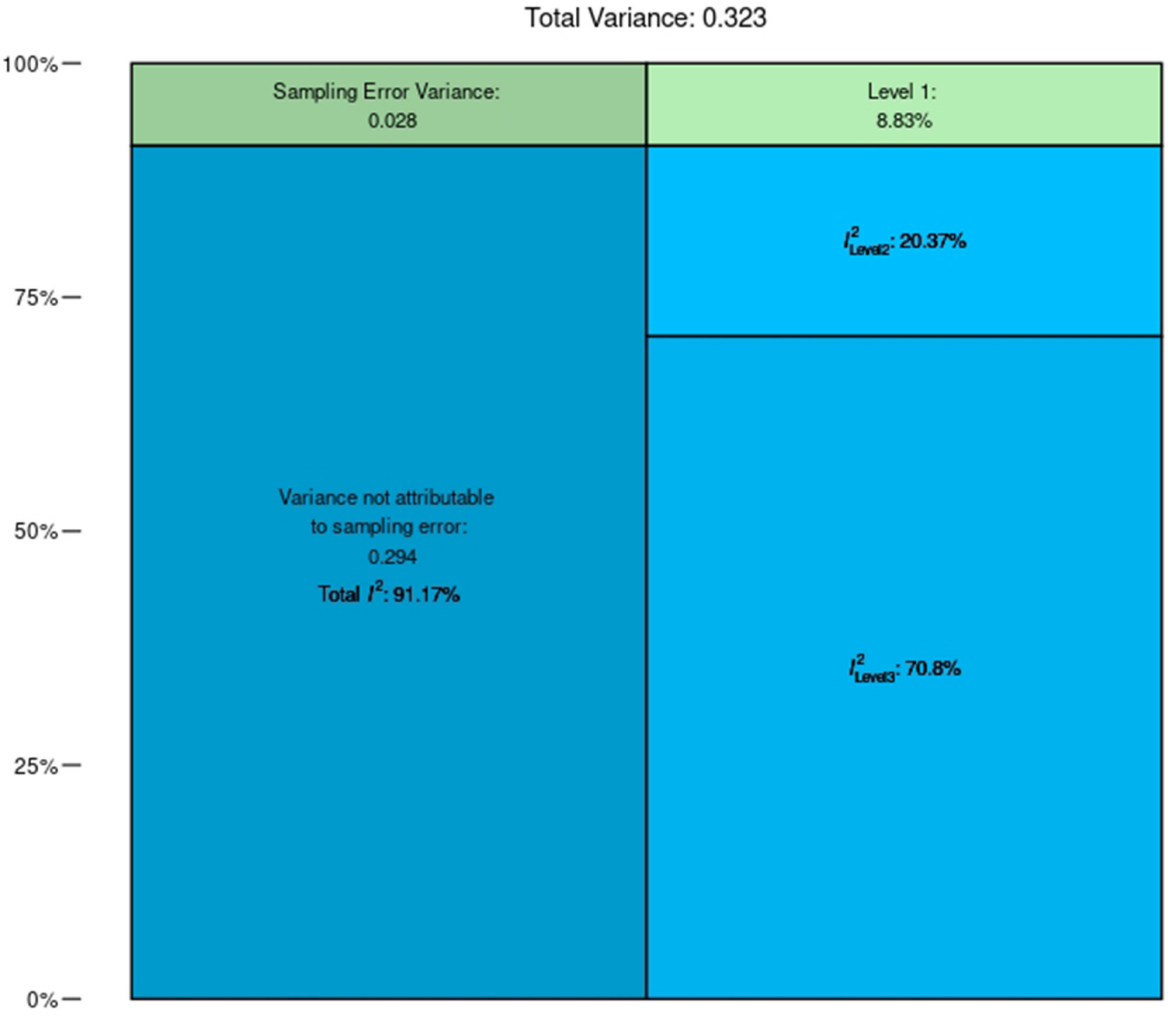
Meta-correlation (Model 1) variance breakdown by level. The majority of heterogeneity within this model results from Level 3.

The initial model stipulated in Equation (1) estimated the average correlation of baseline ability with training gains. Results demonstrated a significant negative correlation between baseline cognitive performance and training gain (β_0_ = –0.4490, z = –4.1584, *p* < 0.0001) (Figure 5). The direction of the correlation is consistent with a compensation effect; individuals who show worse performance prior to training tend to reap greater gains, but the result is inconclusive. A significant amount of heterogeneity between observations was evidenced [Q_(81)_ = 924.5982, *p* < 0.0001; *I*^2^ = 91.17, *p* < 0.0001] indicating a large amount of variability across the collected literature (Higgins and Thompson, 2002). This high level of heterogeneity was expected due to the diversity of training approaches, ages, and populations included in this review, as well as the hypothesized relevance of the training characteristic predictors for this correlation. The multi-level nature of this model allowed for further separation of this heterogeneity across between- and within-study clusters (Nakagawa and Santos, 2012). This break-down indicated that 20.37% of heterogeneity was between clusters (i.e., between individual studies) and the remaining 70.8% was within clusters (i.e., within individual studies) (Figure 6). Complete model results are reported in Table 2.

**Table 2:** Model 1: Meta-correlation results.

***Model Estimates***					
	**Estimate**	**SE**	***p*-value**	**Lower bound**	**Upper bound**
Correlation value	–0.4490	0.1080	<0.0001	–0.661	–0.2374
*Variance*					
	Estimate	Sqrt	Levels		
Level 1: σ^2^	0.0657	0.2564	82		
Level 2: σ^2^	0.2284	0.4479	25		
*Heterogeneity*					
	Q(df)	*I*^2^			
	924.598(81)	91.17			

#### 3.2.3. Model 2: Meta-Regression

As analysis of heterogeneity in the base model indicated a significant amount of variability between observations, meta-regression analyses were conducted. However, the characteristics of the collected eligible correlations did not allow for the original model analyzing both the main effects and interaction between cognitive training domain and cognitive training approach to be carried out. As foreshadowed by prior work, executive function and episodic memory training studies in the present sample perfectly aligned with the use of process and strategy training approaches, respectively (see Table 1).

(3) $$\begin{array}{l} \begin{array}{l} Level1:{\hat{\theta }}_{ij}=\theta_{ij}+\epsilon_{ij}\\ Level2:\theta_{ij}=\kappa_{j}+\xi_{\left(2\right)ij}\\ Level3:\kappa_{j}=\beta_{0}+\beta_{1}\left(DomainApproach\right)+\xi_{\left(3\right)j} \end{array} \end{array}$$

A large amount of residual heterogeneity remained after the addition of the domain/approach predictor [QE_(80)_ = 891.1806, *p* < 0.0001]. There was no evidence for a significant relationship between training domain/approach and the correlation of baseline individual differences with cognitive training gain (β_1_ = –0.3082, z = –0.9537, *p* = 0.3402). The estimated average correlation remained negative and significant (β_0_ = –0.4099, z = –3.5574, *p* = 0.0004). Complete model results are reported in Table 3.

**Table 3:** Model 2: Meta-regression results.

***Model Estimates***					
	**Estimate**	**SE**	***p*-value**	**Lower bound**	**Upper bound**
Correlation value	–0.4099	0.1152	0.0004	–0.6357	–0.1841
Factor value	–0.4490	0.1080	0.3402	–0.9416	0.3520
*Variance*					
	Estimate	Sqrt	Levels		
Level 1: σ^2^	0.0666	0.2580	82		
Level 2: σ^2^	0.2264	0.4758	25		
*Heterogeneity*					
	QM(df)	QE(df)			
	0.9096(1)	891.181(80)			

#### 3.2.4. Model 3: Exploratory Meta-Analytic Correlation of Executive Function Papers Only

Given the paucity of qualifying episodic memory/strategy cognitive training papers, a third exploratory analysis was conducted using only executive function/process cognitive training papers. This analysis evaluated the meta-analytic correlation coefficient for this subset of the retrieved effect sizes using the same equations described in Equation (1). A total of 22 papers with 76 effect sizes were included. Complete model results are reported in Table 4.

**Table 4:** Model 3: Exploratory Meta-correlation results with executive function/process studies only.

***Model Estimates***					
	**Estimate**	**SE**	***p*-value**	**Lower bound**	**Upper bound**
Correlation value	–0.4101	0.0998	<0.0001	–0.6057	–0.2144
*Variance*					
	Estimate	Sqrt	Levels		
Level 1: σ^2^	0.0521	0.2283	76		
Level 2: σ^2^	0.1629	0.4036	22		
*Heterogeneity*					
	Q(df)	*I*^2^			
	676.499(75)	86.51%			

**Figure 7:**
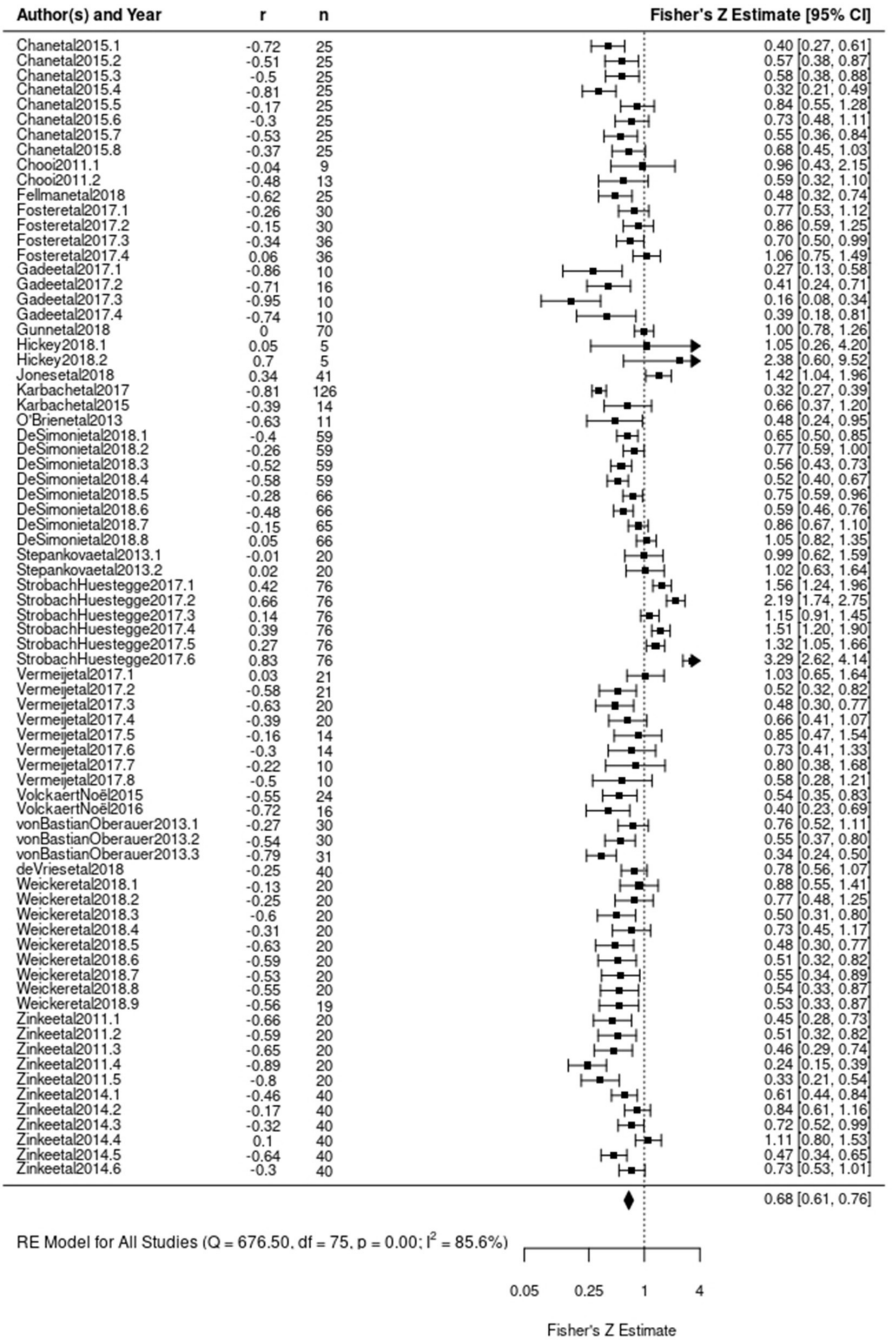
Forest plot of Model 3 results including only executive function trainings. Each correlation coefficient is plotted according to its Fisher's Z transformation. Size of square indicates weight assigned to the observation during analysis. Length of lines extending from square indicates variance associated with observation based on sample size. Each observation's Fisher's Z transformation and confidence interval are presented on the right. The diamond at the bottom indicates the estimated Fisher's Z transformed correlation coefficient derived from Model 3.

Evaluation of publication bias in this restricted dataset demonstrated similar patterns to the complete dataset with significant results in an Egger's test for funnel plot asymmetry (z = –2.5614, *p* = 0.0104). This restricted dataset again demonstrated a significant negative association between baseline ability and gains in that ability as a result of training (β_0_ = –0.4101, z = –4.1074, *p* < 0.0001) with a significant amount of heterogeneity [Q_(75)_ = 676.4997, *p* < 0.0001) (Figure 7).

## 4. Discussion

We highlight three main findings from this systematic meta-analysis of the cognitive training literature. First, baseline individual differences in the cognitive ability trained yield significant compensatory effects. Participants with initially weaker targeted abilities gain more from cognitive training than those who are initially more proficient. This meta-analytic finding is consistent with multiple prior accounts of the influence of individual differences on cognitive training gains, particularly for executive function and process based cognitive training approaches (Lövdén et al., 2012; Karbach and Unger, 2014), and is the first to demonstrate it via a systematic literature synthesis. Second, the literature did not support an interrogation of how this effect might differ based on cognitive domain being trained (executive function vs. episodic memory) and/or training approach (process- vs. strategy-based). Our review retrieved too few eligible papers reporting results of episodic memory or strategy-based training to compare correlations to those of executive function or process-based cognitive trainings—raising questions as to why these papers in particular did not qualify for inclusion. This was accompanied by little variation in the use of process-based approaches with executive function trainings and strategy-based approaches with episodic memory trainings (with a handful of notable exceptions, e.g., Lövdén et al., 2012). Third, despite our sample being predominantly executive function and process-based, a significant amount of heterogeneity was evidenced, suggesting a range of moderators outside of cognitive domain and training approach are at play. We discuss each of these important findings in turn.

### 4.1. A Compensatory Meta-Correlation

Why would the weak gain the most? Cognitive training may close the proficiency gap by providing exposure to the targeted cognitive skill, bolstering individuals' mastery of fundamentals necessary for succeeding with that skill—fundamentals that more advanced participants may have already mastered. Relatedly, compensatory patterns could be the result of ceiling effects, with higher performing individuals having little to no room to improve on existing measures with further cognitive training due to their already high abilities. A ceiling-effect account of compensatory effects could be explored in future systematic reviews that analyze the extent to which effective ceilings were reached across different measures and potential moderating effects of this measurement factor.

Compensatory effects could also arise from participants' expectations about how much they will improve from training, if participants with worse initial abilities hold stronger beliefs that an intervention will be beneficial to them—in comparison to more proficient participants who might feel confident in their existing abilities. This possibility highlights the importance of measuring or manipulating expectations to address their role in training gains (Boot et al., 2013) and their potential interactions with initial ability.

Lastly, compensatory effects could be the result of the specific effect size coefficients extracted for this study: correlation coefficients reflecting the association between baseline ability and gain. Gain scores are most commonly calculated as the difference between performance at test and that original baseline score. The use of this method has been argued to be subject to demonstrating regression to the mean, leading to a greater chance of yielding a compensatory effect (Smoleń et al., 2018). A growing body of work on individual differences in cognitive training gains has moved toward analyzing individual differences effects on cognitive training gains through alternative metrics. These studies test the impact of training via approaches such as latent growth curve modeling or structural equation modeling, which account for variability to support better estimates of the relationship between baseline ability and gain (e.g., Lövdén et al., 2012; Foster et al., 2017; Guye et al., 2017, and others). Given that correlations were the dominant available coefficient from which to calculate a meta-analytic effect, this review highlights the need for both consistency and deeper methodological consideration in future analyses of effects of individual differences in baseline ability on benefits from cognitive training.

### 4.2. Consequences of Eligibility Criteria

There are multiple potential reasons for the difficulty in retrieving eligible papers across our moderators of interest and their combinations. First, our eligibility requirements were stringent. An unexpected difficulty during eligibility screening of retrieved papers was the number of studies utilizing a multi-modal training approach that did not permit for a controlled analysis of the influence of cognitive training in the absence of other approaches (e.g., group therapies, aerobic exercise, and other educational interventions). Future work might include such multi-modal studies to increase the number of usable observations for analysis and more thoroughly represent the current characteristics of the cognitive training literature. While it was important to narrow our scope of inquiry to clearly identify sources of variation as was done here, it will also be valuable to evaluate the possible true effect size of baseline individual differences within the types of studies that are frequently conducted.

Similarly, the eligibility criteria used in this systematic review revealed a paucity of studies meeting requirements for the specific baseline cognition and training gains association being analyzed. Eligibility for inclusion required studies to have investigated the association between individual differences in baseline cognitive ability, with that cognitive ability defined as being of the same type as the one trained, and with cognitive training gains derived from either the final training performance or near transfer performance. This isolated the specific relationship in question to initial ability in cognitive construct “X” and gains in “X” from training in “X.” Many papers were disqualified for deviating from either or both of the baseline cognitive measure and training outcome requirements, highlighting inconsistencies in what these common terms actually represent across the literature. For example, one of the most cited works on this topic analyzed the association of a baseline measure of general fluid intelligence with far transfer outcomes on another fluid intelligence task, after working memory training (Jaeggi et al., 2008). This work is relevant to understanding individual differences in cognitive training, and many papers talk about results such as these in tandem with the types of studies included in this meta-analysis (e.g., Lustig et al., 2009; Lövdén et al., 2012; Karbach and Unger, 2014; Borella et al., 2017; Karbach et al., 2017). However, individual differences in baseline abilities may predict the effectiveness of training in different ways depending on whether baseline, training, and gains target the same ability (e.g., working memory) or target different abilities that require transfer (e.g., training working memory and testing fluid intelligence gains and baseline). Cognitive training could show greater magnification effects in the context of transfer tasks (e.g., Borella et al., 2017; cf. Karbach et al., 2017), given the need to not only acquire a relevant skill but to apply it to another domain, which might require sufficient initial proficiency in that domain. Future work could systematically assess this question, with the challenge of comparing transfer measures that span a wide range of distances from trained cognitive abilities.

### 4.3. Alternative Moderators

While we were unable to sufficiently explore the hypothesized moderators of cognitive domain and training approach, analyses indicated a significant amount of heterogeneity among correlations in both our complete dataset and our exploratory EF-process only dataset. Other moderators not investigated here likely influence whether baseline individual differences predict cognitive training benefits. For example, different age groups respond differently to training, which has been interpreted through the lens of age group differences in cognitive baseline (Lövdén et al., 2012; Bürki et al., 2014; Borella et al., 2017; Karbach et al., 2017). We did not explore age effects within dataset as many observations incorporated multiple age groups (see Supplementary Table 1).

Responsiveness to training is likely also influenced by typical or atypical cognitive status—with specific aspects of an individual's condition potentially impacting the potency of baseline individual differences effects. Many meta-analyses of cognitive training effects limit eligibility to healthy populations for this reason (e.g., Au et al., 2015). Our dataset included a range of atypical populations including individuals with ADD/ADHD, autism, Parkinson's disease and other conditions (see Supplementary Table 1), making it difficult to pose a unified hypothesis for how atypical groups with disparate etiologies might be differently affected by cognitive training when compared to healthy samples. An exploration of typical vs. atypical cognitive status yielded no significant effect on the meta-analytic correlation (β_1_ = -0.1758, z = -0.8930, p < 0.3719), likely because of the small number of atypical observations and the range of conditions within our sample. Future work might focus on the question of how typical vs. atypical groups' baseline abilities influence cognitive training outcomes within a specific condition (e.g., ADD/ADHD) to probe the nuances of magnification or compensation effects based on specific patient characteristics.

Outside of participant characteristics, compensation and magnification effects may also vary based on characteristics of cognitive trainings, such as the specific cognitive components trained. The present dataset, for example, included trainings of distinct executive function processes. Because of the small number of non-working memory executive functions trainings retrieved we were not able to examine the influence of specific executive function effects within the present dataset. Our work sought to determine the meta-analytic effect most representative of existing studies including analysis of baseline individual differences effects on cognitive training outcomes without regard for specific executive function components. This was done to be consistent with the prevailing discussion on the nature of these effects—which typically does not distinguish between specific executive function components. Future work should delve into the potential differences that might arise in this effect between different types of executive functions, as has been done in meta-analytic work looking at the overall effect of cognitive training specific to component types (e.g., Au et al., 2015).

Lastly, the overall potency of a cognitive training (i.e., how effective that training is compared to an active control) could influence who benefits from that training, with weaker interventions tending to benefit those who need them most and stronger interventions showing more equitable benefits. Heterogeneity in the present sample might be driven by moderating effects of any of these factors. While our investigation sought to clarify the pattern of compensation and magnification results seen across executive function and episodic memory training studies using process- and strategy-based approaches, an important direction for future work will be to systematically test the role of these other types of factors to more fully understand to what extent baseline abilities predict benefits from cognitive training.

### 4.4. Recommendations for Future Work

The availability of data limited both the number of eligible papers and the type of effect size coefficients that could be extracted for meta-analysis. To aid in future meta-analytic endeavors, our minimum recommendation is that papers include the following in their main or supplementary materials: complete correlation tables by group as well as complete descriptive statistics by group for all recorded measures. Complete correlation tables would aid in extraction of correlation coefficients (as was meta-analyzed in the present paper) and complete descriptive statistics would aid in the calculation of Cohen's d for meta-analysis of group mean comparisons. Explicit separation of both types of information by group (if multiple groups are present) is essential to extracting information pertinent to cognitive training.

Further, the inclusion of open data sets would greatly assist in the pursuit of meta-analytic work. Within the context of the present study, open datasets would allow for calculation of gain scores when otherwise eligible papers did not report results in this manner. Moving forward, they would allow for the additional calculation of alternative measures and effect sizes not reported in a specific paper. For example, open data sets could support the calculation of indices designed to account for ceiling effects, such as scaling gain scores by dividing them by how much an individual could have improved (i.e., the difference between the maximum scoring of the test and the score obtained by the individual at baseline). In addition, in cases where studies include treatment and control groups, open datasets could allow for *post-hoc* calculation of treatment effects controlling for baseline covariates. For example, baseline scores could be included as a covariate in an ANCOVA model, with group as an independent variable and outcome score as the dependent variables, to account for individual differences in cognitive training effects (Bonate, 2000; Nunes et al., 2011). Open data sets could further support contemporary recommendations for the investigation of individual differences in cognitive training effects including the use of latent variables (e.g., Lövdén et al., 2012; von Bastian and Oberauer, 2014; Guye et al., 2017) (for extensive discussion see Smoleń et al., 2018).

## 5. Conclusion

Using a focused, systematic approach to evaluate the literature, the present meta-analysis demonstrates evidence for a compensatory effect of initial baseline ability on cognitive training gains, suggesting that those best positioned to benefit from cognitive trainings are those who are relatively weaker in ability. Despite finding that few episodic memory or strategy-based studies met our stringent eligibility criteria, preventing us from evaluating the influence of cognitive domain and training type, we found that a substantial amount of heterogeneity still remained among our primarily executive function and process-based studies. This indicates that even in this refined sample, other pertinent moderators of the baseline individual differences effect are at play. The prolific complexity in this field was evident in this study. Future work should tackle defining the problem space of individual differences in initial ability and cognitive training gains. What initial abilities are we interested in—those directly related to the cognitive skill being trained, those nearly related, or those with a far relationship? The same question applies for change after cognitive training. What are the most productive definitions of cognitive training to use—the narrow ones implemented here or broader definitions including multi-modal trainings? What are the most appropriate means of capturing these questions statistically? The present meta-analysis indicates a productive direction forward in targeting cognitive training toward lower performing individuals, and reaffirms the urgency of continuing systematic work on this topic.

## Data Availability Statement

The original contributions presented in the study are included in the article/Supplementary Material, further inquiries can be directed to the corresponding author/s.

## Author Contributions

HT conceived of the idea with feedback from YM. HT and RG carried out data collection. HT performed all analyses. All authors discussed the results and interpretation. HT took the lead in writing the manuscript. YM provided critical revisions. RG provided input. All authors gave final approval of the manuscript.

## Conflict of Interest

The authors declare that the research was conducted in the absence of any commercial or financial relationships that could be construed as a potential conflict of interest.
