# Supplementary material for: Why Does Cognitive Training Yield Inconsistent Benefits? A Meta-Analysis of Individual Differences in Baseline Cognitive Abilities and Training Outcomes
Source: Front Psychol. 2021 May 26;12:662139. doi: 10.3389/fpsyg.2021.662139 (PMC8187947; doi:10.3389/fpsyg.2021.662139)
Supplement: Supplementary file 1 [file Data_Sheet_1.PDF]

Supplementary Material

1 SUPPLEMENTARY ANALYSIS REPORTS

Supplementary analysis reports available at: hilary-traut.shinyapps.io/20201120_tpfkam_dash

2 SUPPLEMENTARY TABLES AND FIGURES

2.1 Tables

Table S1. Extended description of data included in meta-analysis, including: Author (Year) of publication, correlation ID for correlation within paper, training domain (executive function, EF; episodic memory, EP), training approach (process or strategy), age group (children, CH; young adult, YA; older adult, OA), baseline measure (name of baseline task measure), outcome measure (name of outcome measure), n (sample size), and r (correlation coefficient). Cells containing ” indicate information is identical to that above.

Abbreviations: EF = executive function; EP = episodic memory; WM = Working Memory
CH = Child (0 – 18yo); YA = young adult (19 – 54); OA = older adult (55 and up).
+ Training descriptions are limited to training groups within a study eligible for analysis. Additional training groups not eligible are not described here.
* Indicates correlation was multiplied by -1 for interpretability purposes in analysis.

Table S1: List of all observations used in reported analyses.

Author (Year)	ID	Training Domain	Training Approach	Training Group	Population	Age Group	Baseline Measure	Outcome Measure	n	r
Chan et al. (2015)	1	EF	process	WM	Typical	YA, OA	2-back ACC spatial n-back	2-back ACC spatial n-back	25	-0.72
”	2	”	”	”	”	”	3-back ACC spatial n-back	3-back ACC spatial n-back	25	-0.51
”	3	”	”	”	”	”	4-Back ACC spatial n-back	4-Back ACC spatial n-back	25	-0.5
”	4	”	”	”	”	”	5-back ACC spatial n-back	5-back ACC spatial n-back	25	-0.81
”	5	”	”	”	”	”	2-back RT spatial n-back	2-back RT spatial n-back	25	-0.17
”	6	”	”	”	”	”	3-back RT spatial n-back	3-back RT spatial n-back	25	-0.3
”	7	”	”	”	”	”	4-Back RT spatial n-back	4-Back RT spatial n-back	25	-0.53
”	8	”	”	”	”	”	5-back RT spatial n-back	5-back RT spatial n-back	25	-0.37
Chooi (2011)	1	EF	process	WM - 2-week training group	Typical	YA	OSPAN	OSPAN	9	-0.036

Continued on next page

Table S1 – continued from previous page

Author (Year)	ID	Training Domain	Training Approach	Training Group	Population	Age Group	Baseline Measure	Outcome Measure	n	r
”	2	”	”	WM - 5-week training group	”	YA, OA	”	”	13	-0.48
Fellman et al. (2018)	1	EF	process	WM	Parkinson Disorder	YA, OA	WM composite - n-back, SUS, forward digit span	WM composite - n-back, SUS, forward digit span	25	-0.62
Foster et al. (2017)	1	EF	process	WM - Complex span training group	Typical	YA	Complex span task (reading span)	Complex span task (reading span)	30	-0.26
”	2	”	”	WM - Complex span training group	”	”	Complex span task (rotation span)	Complex span task (rotation span)	30	-0.15
”	3	”	”	WM - Running span training group	”	”	Running span task (running span images)	Running span task (running span images)	36	-0.34
”	4	”	”	WM - Running span training group	”	”	Running span task (running span arrows)	Running span task (running span arrows)	36	0.06
Gade, Zoelch, & Seitz-Stein (2017)	1	EF	process	WM - Study 1 training group	Typical	CH	Corsi Block Task	Corsi Block Task	10	-0.86

Continued on next page

Table S1 – continued from previous page

Author (Year)	ID	Training Domain	Training Approach	Training Group	Population	Age Group	Baseline Measure	Outcome Measure	n	r
”	2	”	”	WM - Study 2 training group	”	”	”	”	16	-0.71
”	3	”	”	WM - Study 3 training group	”	”	”	”	10	-0.95
”	4	”	”	WM - Study 4 training group	”	”	”	”	10	-0.74
Gunn et al. (2018)	1	EF	process	WM	Alcohol Use Disorder; Typical	YA	OSPAN	OSPAN	70	-0.01
Hickey (2018)	1	EF	process	WM (Cogmed)	Typical	CH	AWMA Verbal WM Composite - counting recall, listening recall	Cogmed WM Training improvement-index	5	0.05
”	2	”	”	”	”	”	AWMA? Visuo-spatial WM Composite - odd one out, spatial recall	”	5	0.7
Jones et al. (2018)	1	EF	process	WM	ADHD	CH	Working Memory	Working Memory	41	0.34
Karbach, Konen, & Spengler (2017)	1	EF	process	Task Switching	Typical	CH, YA, OA	Task-switching paradigm	Task-switching paradigm	126	0.81*
Karbach, Strobach, & Schubert (2015)	1	EF	process	WM	Typical	CH	WM adaptive span task	WM adaptive span task	14	-0.39

Continued on next page

Table S1 – continued from previous page

Author (Year)	ID	Training Domain	Training Approach	Training Group	Population	Age Group	Baseline Measure	Outcome Measure	n	r
Lövdén et al. (2012)	1	EP	strategy	Method of Loci	Typical	CH	Episodic Memory Test	Episodic Memory Test	50	-0.85
”	2	”	”	”	”	YA	”	”	29	-0.99
”	3	”	”	”	”	OA	”	”	29	-0.9
McKitrick, Friedman, & Brooks (1999)	1	EP	strategy	Mnemonic instruction - Method of Loci	Typical	OA	Serial word recall	Serial word recall	224	-0.33
”	2	”	”	”	”	”	Name recall	Name recall	224	-0.44
O'Brien et al. (2013)	1	EF	process	Selective Attention	Typical	OA	Visual search task	Visual search task	11	-0.63
De Simoni & von Bastian (2018)	1	EF	process	WM - Updating training group	Typical	YA	Digits Updating Task	Digits Updating Task	59	-0.4
”	2	”	”	”	”	”	Locations Updating Task	Locations Updating Task	59	-0.26
”	3	”	”	”	”	”	Arrows Updating Task	Arrows Updating Task	59	-0.52
”	4	”	”	”	”	”	Letters Updating Task	Letters Updating Task	59	-0.58
”	5	”	”	WM - Binding training group	”	”	Symbol-Digit Binding Task	Symbol-Digit Binding Task	66	-0.28
”	6	”	”	”	”	”	Noun-Verb Binding Task	Noun-Verb Binding Task	66	-0.48

Continued on next page

Table S1 – continued from previous page

Author (Year)	ID	Training Domain	Training Approach	Training Group	Population	Age Group	Baseline Measure	Outcome Measure	n	r
”	7	”	”	”	”	”	Color-Location Binding Task	Color-Location Binding Task	65	-0.15
”	8	”	”	”	”	”	Fractal-Location	Fractal-Location	66	0.05
Singer, Lindenberger, & Baltes (2003)	1	EP	strategy	Method of Loci	Typical	OA	Episodic Memory Test	Episodic Memory Test	96	0.14
Stepankova et al. (2013)	1	EF	process	WM - 10 session training group	Typical	OA	Adaptive n-back	Adaptive n-back	20	-0.01
”	2	”	”	WM - 20 session training group	”	”	”	”	20	0.02
Strobach & Huestegge (2017)	1	EF	process	WM - Updating	Typical	YA, OA	Memory Interrupted	Working Memory	76	0.42
”	2	”	”	”	”	”	Trail Making Test 2	Trail Making Test 2	76	0.66
”	3	”	”	”	”	”	Shuffler	Shuffler	76	0.14
”	4	”	”	”	”	”	Restorer	Restorer	76	0.39
”	5	”	”	”	”	”	Turning Tops	Turning Tops	76	0.27
”	6	”	”	”	”	”	Trail Making Test 1	Trail Making Test 1	76	0.83
Vermeij et al. (2017)	1	EF	process	WM - Cogmed	Typical	OA	WM - n-back (0-back)	WM - n-back (0-back)	21	0.03

Continued on next page

Table S1 – continued from previous page

Author (Year)	ID	Training Domain	Training Approach	Training Group	Population	Age Group	Baseline Measure	Outcome Measure	n	r
”	2	”	”	”	”	”	WM - n-back (1-back)	WM - n-back (1-back)	21	-0.58
”	3	”	”	”	”	”	WM - n-back (2-back)	WM - n-back (2-back)	20	-0.63
”	4	”	”	”	”	”	WM - n-back (3-back)	WM - n-back (3-back)	20	-0.39
”	5	”	”	”	Mild Cognitive Impairment	”	WM - n-back (0-back)	WM - n-back (0-back)	14	-0.16
”	6	”	”	”	”	”	WM - n-back (1-back)	WM - n-back (1-back)	14	-0.3
”	7	”	”	”	”	”	WM - n-back (2-back)	WM - n-back (2-back)	10	-0.22
”	8	”	”	”	”	”	WM - n-back (3-back)	WM - n-back (3-back)	10	-0.5
Volckaert & Noël (2015)	1	EF	process	Inhibition	Typical	CH	Inhibition composite - Traffic Light, Cat–Dog–Fish Stroop, HTKS	Inhibition composite - Traffic Light, Cat–Dog–Fish Stroop, HTKS	24	-0.55

Continued on next page

Table S1 – continued from previous page

Author (Year)	ID	Training Domain	Training Approach	Training Group	Population	Age Group	Baseline Measure	Outcome Measure	n	r
Volckaert & Noël (2016)	1	EF	process	Inhibition	Externalizing Behavioral Problems	CH	Inhibition composite - Traffic Light, Cat–Dog–Fish Stroop, HTKS	Inhibition composite - Traffic Light, Cat–Dog–Fish Stroop, HTKS	16	-0.72
von Bastian & Oberauer (2013)	1	EF	process	WM - Storage-processing training group	Typical	YA	WM - Brown Peterson	WM - Brown Peterson	30	-0.27
”	2	”	”	WM - Relational integration training group	”	”	WM - relational integration	WM - relational integration	30	-0.54
”	3	”	”	WM - Supervision training group	”	”	WM - Task Switching	WM - Task Switching	31	-0.79
de Vries et al. (2018)	1	EF	process	WM - Adaptive switching training group	Autism	CH	BRIEF EF composite	WM/Flexibilit Training	40	-0.25
Weicker et al (2018)	1	EF	process	WM - WOME	Typical	OA	WM - digit span backwards	WM - digit span backwards	20	-0.13
”	2	”	”	”	”	”	WM - digit span forwards	WM - digit span forwards	20	-0.25

Continued on next page

Table S1 – continued from previous page

Author (Year)	ID	Training Domain	Training Approach	Training Group	Population	Age Group	Baseline Measure	Outcome Measure	n	r
”	4	”	”	”	”	”	WM - span board backwards	WM - span board backwards	20	-0.6
”	5	”	”	”	”	”	WM - span board forwards	WM - span board forwards	20	-0.31
”	6	”	”	”	”	”	WM - PASAT 2 seconds	WM - PASAT 2 seconds	20	-0.63
”	7	”	”	”	”	”	WM - PASAT 3 seconds	WM - PASAT 3 seconds	20	-0.59
”	8	”	”	”	”	”	WM - spatial addition	WM - spatial addition	20	-0.53
”	9	”	”	”	”	”	WM - symbol span	WM - symbol span	20	-0.55
”	10	”	”	”	”	”	WM - ospan	WM - ospan	19	-0.56
Zinke et al. (2011)	1	EF	process	WM	Typical	OA	Working Memory Capacity (Digit Span Forward)	Digit Span Forward	20	-0.66
”	2	”	”	”	”	”	Working Memory Capacity (Digit Span Backwards)	Digit Span Backwards	20	-0.59
”	3	”	”	”	”	”	Working Memory Capacity (Corsi Block Forward)	Corsi Block Forward	20	-0.65

Continued on next page

Table S1 – continued from previous page

Author (Year)	ID	Training Domain	Training Approach	Training Group	Population	Age Group	Baseline Measure	Outcome Measure	n	r
”	4	”	”	”	”	”	Working Memory Capacity (Corsi Block Backward)	Corsi Block Backwards	20	-0.89
”	5	”	”	”	”	”	Working Memory Capacity (K-ABC)	K-ABC (VSWM)	20	-0.8
Zinke et al. (2014)	1	EF	process	EF	Typical	OA	Visuospatial WM (Picture grid)	Visuospatial WM (Picture grid)	40	-0.46
”	2	”	”	”	”	”	”	Visuospatial WM (Block span)	40	-0.17
”	3	”	”	”	”	”	Verbal WM (Subtract-2)	Verbal WM (Subtract-2)	40	-0.32
”	4	”	”	”	”	”	”	Verbal WM (Letter span plus)	40	0.1
”	5	”	”	”	”	”	Executive control (Tower of London)	Executive control (Tower of London)	40	-0.64
”	6	”	”	”	”	”	”	Executive control (Tower of Hanoi)	40	-0.3
